# Immunomodulatory role of mesenchymal stem cell therapy in liver fibrosis

**DOI:** 10.3389/fimmu.2022.1096402

**Published:** 2023-01-04

**Authors:** Peng Liu, Yerong Qian, Xin Liu, Xulong Zhu, Xufeng Zhang, Yi Lv, Junxi Xiang

**Affiliations:** ^1^ Center for Regenerative and Reconstructive Medicine, Med-X Institute, First Affiliated Hospital of Xi’an Jiaotong University, Xi’an, Shaanxi, China; ^2^ Department of Hepatobiliary Surgery, First Affiliated Hospital of Xi’an Jiaotong University, Xi’an, Shaanxi, China; ^3^ Department of Radiotherapy, Xi’an Medical University, Xi’an, Shaanxi, China; ^4^ Department of Surgical Oncology, Shaanxi Provincial People’s Hospital, Xi’an, Shaanxi, China

**Keywords:** liver fibrosis, mesenchymal stem cell, immunomodulatory effects, exosome, antifibrosis

## Abstract

Liver fibrosis is a fibrogenic and inflammatory process that results from hepatocyte injury and is characterized by hepatic architectural distortion and resultant loss of liver function. There is no effective treatment for advanced fibrosis other than liver transplantation, but it is limited by expensive costs, immune rejection, and postoperative complications. With the development of regenerative medicine in recent years, mesenchymal stem cell (MSCs) transplantation has become the most promising treatment for liver fibrosis. The underlying mechanisms of MSC anti-fibrotic effects include hepatocyte differentiation, paracrine, and immunomodulation, with immunomodulation playing a central role. This review discusses the immune cells involved in liver fibrosis, the immunomodulatory properties of MSCs, and the immunomodulation mechanisms of MSC-based strategies to attenuate liver fibrosis. Meanwhile, we discuss the current challenges and future directions as well.

## 1 Introduction

Liver fibrosis is a complex fibrogenic and inflammatory process that results from chronic liver injury. The primary pathophysiology of liver fibrosis is increased collagen deposition of types I and III in the extracellular matrix (ECM) (1, 2). Major etiological agents of liver fibrosis include alcohol, viruses, metabolic, and congenital disorders, all of which can lead to hepatocyte injury and hepatic stellate cell (HSC) activation, resulting in excessive ECM deposition and structural disorders in the liver (3, 4). Despite distinct pathogenesis, their common outcome is the development of liver cirrhosis. In recent years, the incidence and mortality of hepatic fibrosis have increased steadily and become a substantial global health burden. Other than liver transplantation, there is no curative treatment for end-stage cirrhosis. However, expensive costs, immune rejection, and postoperative complications limit liver transplantation (5, 6).

Stem cell transplantation has emerged as the most promising treatment for liver fibrosis since the advent of regenerative medicine (7–9). Stem cells are a population of cells with self-renewal, proliferation, and pluripotent differentiation potential that can differentiate into multiple cell types under defined conditions (10, 11). Embryonic stem cells (ESCs) (12, 13), induced pluripotent stem cells (iPSCs) (14–17), and mesenchymal stem cells (MSCs) (18–22) have been investigated the most in the treatment of liver fibrosis. ESCs, the prototype of pluripotent stem cells, have nearly unlimited self-renewal capacity and differentiation potential (23). iPSCs display similar surface antigens expression, proliferation capacity, morphology, and gene expression characteristics as embryonic stem cells (24). ESCs and iPSCs achieve therapeutic effects mainly by differentiating into mature hepatocytes *in vitro* or *in vivo (* 8). MSCs, on the other hand, not only have the potential for self-renewal and differentiation (25) and can regulate the immune response (7). Moreover, since abnormal immune responses are the primary cause of liver fibrosis, MSCs are the most promising stem cells for treating liver fibrosis, as they can slow or even reverse the progression of liver fibrosis (26–28).

This review focuses on the immune cells involved in liver fibrosis, the immunomodulatory properties of MSCs, and the immunomodulation mechanisms of MSC-based strategies to attenuate liver fibrosis. The challenges and future directions were also discussed.

## 2 Immune cells participate in the process of liver fibrosis

According to common knowledge, the liver is composed of primary hepatocytes, cholangiocytes, Kupffer cells (KCs), liver sinusoidal endothelial cells (LSECs), HSCs, fibroblasts, lymphocytes, oval cells, lymphocytes, and other immune cells (29). The liver’s blood supply originates from the hepatic artery and portal vein and passes through infections or toxins of systemic and intestinal origin. Hence, the liver is highly susceptible to pathogens that cause acute or chronic liver injury (30). Injury to intrahepatic parenchymal cells, such as hepatocytes or cholangiocytes, causes liver fibrosis. Varied etiologies could contribute to liver damage, including inflammation, chronic viral hepatitis, alcohol consumption, chronic cholestasis, and non-alcoholic steatohepatitis (NASH). Although the etiologies are different, the initial phase often includes hepatocyte injury, which subsequently causes the release of oxygen radicals and inflammatory molecules. These pro-inflammatory mediators stimulate KCs and hepatic sinusoidal endothelial cells, leading to the transdifferentiation of HSCs from a quiescent to an activated phenotype (1–3). In addition, other liver-specific immune cells, including natural killer (NK) cells, natural killer T cells (NKT) cells, dendritic cells (DCs), and neutrophils, react to injured hepatocytes by producing cytokines and initiating inflammatory responses. These inflammatory cytokines (such as transforming growth factor-β (TGF-β) and platelet-derived growth factor (PDGF)) could stimulate and transdifferentiate HSCs into myofibroblasts (3, 4). HSCs are the most critical non-parenchymal cells of the liver, located in the Disse space, and play a crucial role in developing liver fibrosis. Fibroblasts in the bone marrow or circulating blood, as well as hepatocytes and cholangiocytes, can also be transdifferentiated into myofibroblasts by epithelial-mesenchymal transition (EMT). However, those derived from activated HSCs are the most common (4).

**Figure 1:**
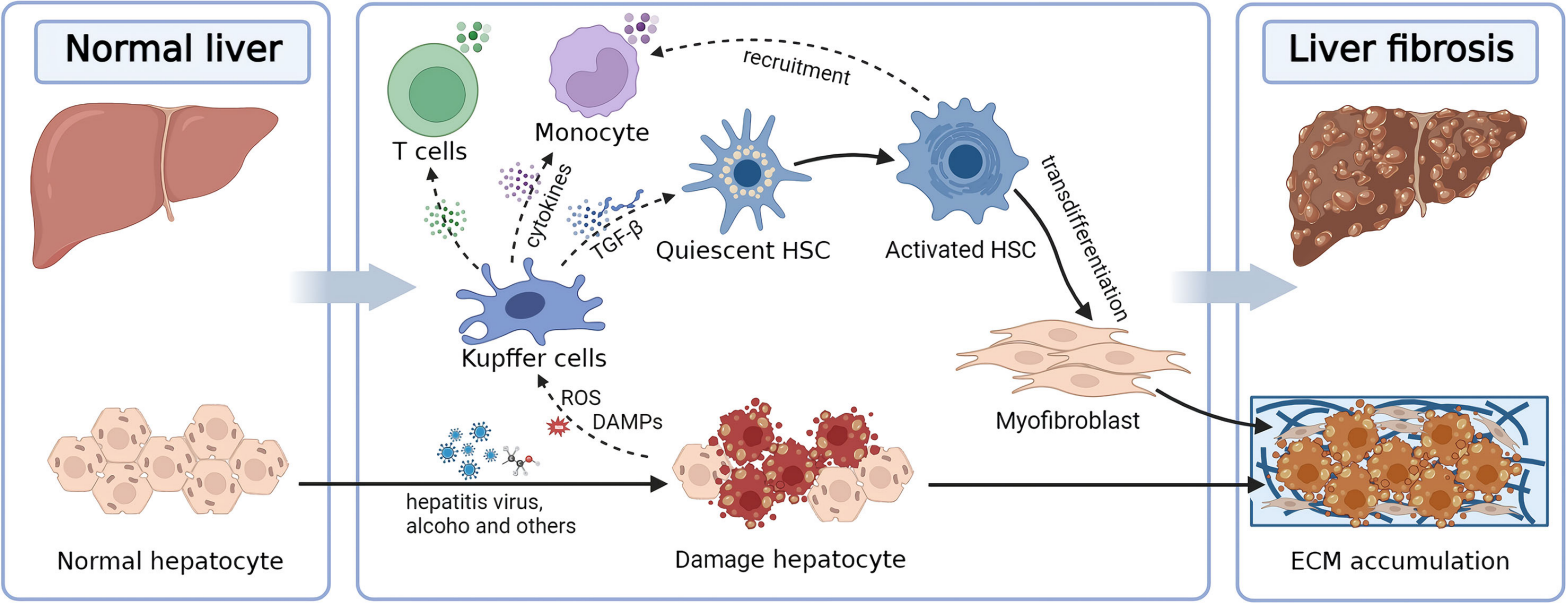
Diagram illustrating the pathological mechanisms underlying liver fibrosis. Hepatic fibrosis is induced by the imbalance between ECM production and degradation, driven by HSCs activated by hepatocyte injury. ECM, extracellular matrix; HSC, hepatic stellate cell; TGF-β, transforming growth factor-β.

Activation of HSCs results in the generation of a massive ECM and the expression of α-smooth muscle actin (α-SMA). TGF-β is the primary cytokine involved in the activation of HSC transdifferentiation and the EMT signal. TGF-β1 activation increases ECM synthesis and inhibits ECM degradation, thereby accelerating the liver fibrosis development. In addition, myofibroblasts are known to produce tissue inhibitors of matrix metalloproteinases (TIMPs) to prevent matrix metalloproteinases (MMPs) from degrading the ECM and maintaining ECM integrity (**Figure 1**) (31, 32).

## 3 The immunomodulatory properties of mesenchymal stem cells

MSCs were initially utilized primarily for tissue repair and regeneration. Subsequently, they have been increasingly used to treat graft-versus-host disease (GVHD) (33) and autoimmune diseases like lupus (34) and Crohn’s disease (35). Furthermore, the clinical potential of MSCs has been extended to treat myocardial infarction (36), stroke (37, 38), multiple sclerosis (39), liver cirrhosis (18, 40), diabetes (41), lung injuries (42, 43), and cancer (44). MSCs have been isolated and expanded from numerous adult and perinatal tissues, including bone marrow, adipose tissue, peripheral blood, fetal tissues, dental pulp, umbilical cord, and placental tissues (45).

Currently, it is believed that MSC must possess at least the following three characteristics (46): first, the cells must be able to grow on plates; second, CD90, CD73, and CD105 must be expressed, while HLA class II, CD19 or CD79a and CD14, CD34, CD45 or CD11b are negative; and third, the cells must be able to differentiate into osteoblasts, chondrocytes, and adipocytes.

MSCs are multipotent cells emerging as the most promising method of allogeneic cell treatment (45). MSCs have natural immunomodulatory properties, trophic capacities, and strong *in vitro* self-renewal capacity, and their immune-modulatory actions can be easily manipulated (44). MSCs influence most immune cell functions through direct contact and factors in the local microenvironment. Previous research has revealed that cytokines released by MSCs are primarily responsible for the immunomodulatory actions of MSCs (28). However, recent research has shown that apoptotic and metabolically inactive MSCs still possess the immunomodulatory capability, with regulatory T cells and monocytes playing a vital role (47).

MSCs regulate both innate and adaptive immunity (47), and their immunomodulatory functions are predominantly exerted through cell-to-cell contact and paracrine activity with macrophages, monocytes, neutrophils, T cells, B cells and natural killer (NK) cells. By secreting prostaglandin E2, MSCs convert pro-inflammatory M1 into anti-inflammatory M2 macrophages (PGE2) (48). MSCs can also modulate immune responses by activating the Notch 1 signaling pathway, releasing HLA-G5, PGE2, and TGF-1, and boosting the activation and proliferation of CD4+CD25+FoxP3+ regulatory T cells (Tregs) (49). MSCs decrease the proliferative potential of CD8+ T lymphocytes by producing indoleamine 2,3-dioxygenase (IDO) and heme oxygenase-1 (HO-1) and increase the rate of CD4+ T lymphocytes changing from type 1 T helper (TH1) to TH2 phenotype (50). In addition, hepatocyte growth factor (HGF) and IL-6 produced by MSCs, for instance, prevent the differentiation of monocytes into dendritic cells and reduce their propensity to cause inflammation, reduce the secretion of pro-inflammatory cytokines IL-12 and IFN-γ, and increase the production of anti-inflammatory cytokine IL-10, thereby inhibiting T-cell activation (51). MSCs reduce the generation of the pro-inflammatory cytokine TNF by inhibiting the activity of blast cells.

Successful immunomodulation and tissue regeneration depend on the interplay between MSCs and macrophages, especially juxtacrine mechanisms and cell–cell contact (52). Inflammatory factors released by M1 macrophages or activated T cells stimulate MSCs to secrete cytokines to differentiate monocytes toward the M2 type of the anti-inflammatory phenotype (53). It is known that MSCs may release anti-inflammatory or pro-inflammatory cytokines (such as IL1b, IL-6, IL-8, and IL-9) to mediate their immunomodulatory effects (54). As stated above, MSCs either inhibit or promote inflammation based on their exposure to pathological circumstances. The ultimate immunomodulatory effect may depend on the ratio of anti-inflammatory to pro-inflammatory factors in their surrounding environment (55).

## 4 Mesenchymal stem cell therapies for liver cirrhosis: Immune regulation plays a central role

### 4.1 Potential mechanisms of MSC-based treatment of liver fibrosis

Many studies have investigated the mechanisms of MSCs in the treatment of liver fibrosis from diverse perspectives, and they have been classified into three types (13, 22, 56). (1) MSCs can transdifferentiate into hepatocytes or merge with existing hepatocytes when introduced into injured liver tissue, making them a valuable resource for liver tissue regeneration and repair; (2) MSCs are capable of producing various cytokines, growth factors, and exosomes, which stimulate the regeneration of damaged liver tissue; (3) MSCs possess inhibitory effects on several other cell types, including NKs, B and T lymphocytes, allowing them to exercise immunomodulatory effects on liver diseases.

By migrating to damaged tissues, transplanted MSCs contribute to the regeneration of the damaged liver *via* hepatocyte differentiation mechanisms. Adding specific growth factors to *in vitro* culture can promote the differentiation of MSCs into hepatocyte-like cells with liver-specific morphology and functions, such as uptake of low-density lipoprotein and indocyanine green, secretion of albumin and urea, glycogen storage, and cytochrome P450 activity (25, 57). For instance, intrasplenic-grafted MSCs transplanted into liver tissue treated with carbon tetrachloride (CCl_4_) undergo hepatogenic differentiation into HLCs with typical hepatocyte morphology and form a three-dimensional structure (58). Transplantation of hepatocyte-differentiated MSCs further inhibited hepatocyte necrosis and stimulated liver regeneration, thereby enhancing the survival rate of the ALF model after transplantation into damaged liver tissue.

**Figure 2:**
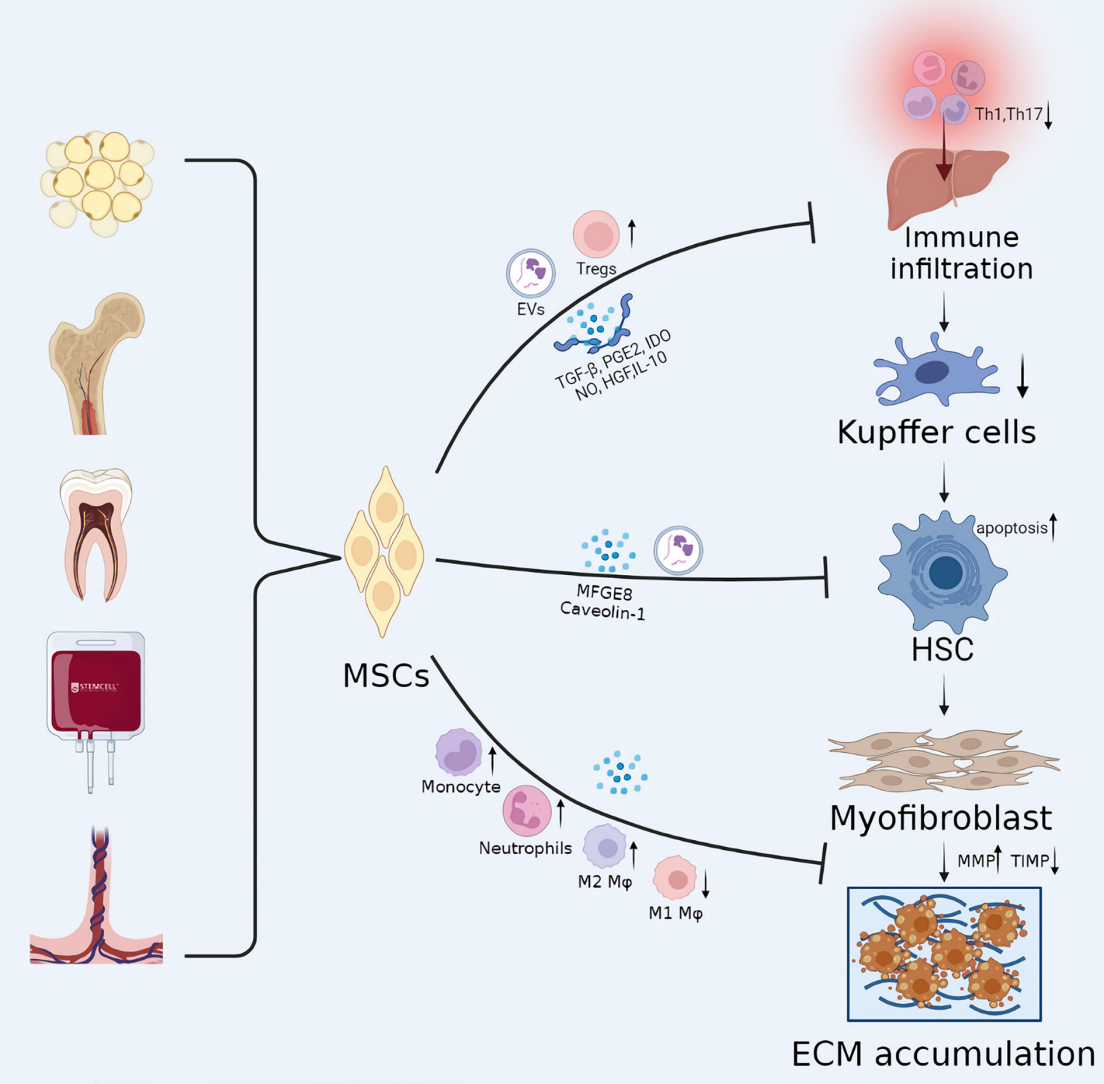
The potential mechanisms of MSCs in liver cirrhosis. The development of liver fibrosis is a wound-healing process involving key events, including hepatocyte injury, immune cell infiltration, HSC activation, and excessive ECM deposition. MSC transplantation can play a therapeutic role in every stage of liver fibrosis, with immunomodulatory effects playing a central role. EVs, extracellular vesicles; Mφ, Macrophages; IDO, indoleamine 2,3-dioxygenase.

However, several studies have demonstrated that transplanted MSCs rarely undergo hepatic differentiation and vanish from the liver within one month (59). These studies indicate that MSCs stimulate liver regeneration *via* immune regulation and paracrine mechanisms (25, 56, 60). By regulating innate and adaptive immune cells, including T lymphocytes, regulatory T cells, helper T cells, B lymphocytes, and regulatory B cells, MSCs create a tolerant environment for maintaining immune homeostasis *in vivo* (61–64). As previously mentioned, the development of liver fibrosis is a wound-healing process involving key events, including hepatocyte injury, immune cell infiltration, HSCs activation, and excessive ECM deposition. Therefore, the immunomodulatory function of MSCs can be exploited to ameliorate liver fibrosis, and it has been an area of intense study interest (**Figure 2**).

### 4.2 MSC can inhibit immune cell infiltration

Infiltration of immune cells is a necessary stage in liver injury. MSCs create an immunological-tolerant milieu in liver tissue by reducing the infiltration of pro-inflammatory immune cells and promoting the recruitment of anti-inflammatory immune cells, preventing acute or chronic liver damage. Producing soluble factors, including TGF-β, PGE2, IDO, NO, and HGF, MSCs were able to inhibit the activation of T cells. MSCs can induce the transdifferentiation of CD4+ T cells into CD25+Foxp3+ regulatory T cells (Tregs) through the release of TGF-β (7, 49). Studies have demonstrated that MSCs dramatically decreased the number of CD4+ T cells invading the liver, the proportion of activated CD4+ T lymphocytes, and the total concentration of Th1 cells, and subsequently induced regulatory DCs and Tregs in the liver to ameliorate liver damage (65). MSCs significantly alleviated CCl_4_-mediated liver fibrosis by decreasing the proportion of Th17 cells and increasing the levels of CD4+IL-10+ T cells and immunosuppressive factors (including kynurenine, IDO, and IL-10). In addition, MSCs enhanced liver function and ameliorated clinical symptoms in patients with hepatitis B virus-mediated decompensated cirrhosis by significantly downregulating the expression levels of IL-6 and TNF-α, while upregulating the expression level of IL-10 (66).

### 4.3 Blocking HSC is a key target for MSC to attenuate liver fibrosis

Blocking the activation of HSCs is one of the most crucial intervention targets for liver fibrosis (8). Pro-inflammatory mediators, oxidative stress molecules, and inflammatory stimulants produced by apoptosis or necrosis of liver parenchymal cells initiate the activation of HSCs (4). Activated HSCs subsequently release a variety of inflammatory chemicals that enhance the liver’s inflammatory response. TGF-β is considered to be one of the most important signaling molecules for the activation of HSCs.

According to certain studies, milk fat globule-EGF factor 8 (MFGE8), one of the mediators secreted by MSC, is an anti-fibrotic protein that prevents the activation of HSCs by suppressing TGF-β type I receptor (TGFBR1) (67). Moreover, Caveolin-1, another possible target for MSC therapy, inhibited HSCs significantly (68). The expression of Wnt pathway-related proteins such as PPARγ, Wnt3a, Wnt10b, β-catenin, and WISP1 and Cyclin D1 is also known to be critical for HSC activation, and MSC has inhibitory effects on several molecules of this pathway (19). Ohara et al. (69) demonstrated that amniotic membrane-derived MSCs (AMSCs) could inhibit HSCs activation by downregulating the upstream steps of the LPS/TLR4 signaling pathway without inhibiting downstream NF-κB transcriptional activity. Qiao et al. (70) proved that hBM-MSCs significantly inhibited the proliferation of activated HSCs by inducing the apoptotic process of activated HSCs. Moreover, hBM-MSCs decreased the expression of peroxisome proliferator-activated receptor γ and α1(I) collagen and α-smooth muscle actin (α-SMA) in activated HSCs by decreasing the signalling pathway of NADPH oxidase, thereby delaying the progression of liver fibrosis.

### 4.4 MSC’s function in ECM degradation and remodeling

As mentioned above, liver fibrosis is associated with excessive ECM deposition and decreased ECM lytic activity. ECM degradation and remodelling are considered vital targets for reversing liver fibrosis and delaying the progression of liver fibrosis. Modulating TGF-β signalling is one of the important mechanisms of MSC-based modulation of liver fibrosis (8). MSCs participate in the regulation of TGF-β downstream pathways. MSCs were able to significantly down-regulate the mRNA expression of TGF-β1 and TGFBR1 downstream molecule SMAD3 and increase the mRNA expression of SMAD7 (71). It has been demonstrated that SMAD3 upregulates the expression of the pro-fibrotic factor α-SMA or Col1a1, whereas SMAD7 has an anti-fibrotic impact. ECM components laminin and hyaluronic acid were significantly decreased when BMSC overexpressed SMAD7.

In addition, it was demonstrated that by overexpressing SMAD7, MSC could enhance serum MMP-1 levels and reduce TIMP-1 levels. MMP-1 is a matrix metalloproteinase that degrades matrix collagen type I (Col1a1) and collagen type III (Col3a1). TIMPs, on the other hand, inhibit MMP activity by forming reversible covalent complexes with the corresponding MMPs. MSCs have been demonstrated to induce the infiltration of host monocytes and neutrophils into the liver and relieve fibrosis *via* MMP release (18). Luo et al. (72) demonstrate for the first time that BM-MSC transplantation promotes activation of MMP13-expressing M2 macrophages and suppresses M1 macrophages, which further inhibit HSCs, which play a synergistic role in attenuating liver fibrosis.

### 4.5 MSC can alleviate liver fibrosis *via* extracellular vehicles

In addition to direct cell-to-cell contact and paracrine cytokines, MSC can ameliorate liver fibrosis *via* extracellular vehicles (EVs). EVs produced by MSCs include exosomes (40-100 nm in diameter) and microvesicles (MVs, 0.1-1 mm in diameter). EVs contribute to the therapeutic potential of MSCs by enhancing intercellular contacts for the transport of paracrine substances during angiogenesis, tissue repair, and immunomodulation (73). Exosomes are nanoscale EVs derived from MVB, secreted into the extracellular microenvironment by the fusion of MVB with the plasma membrane. Exosomes can be taken up by target cells in the local milieu or transported to distant regions *via* biofluids. Exosomes contain numerous cytoplasmic and membrane proteins, such as nucleic acids (miRNA, mRNA, dsDNA, ssDNA, and mtDNA), ECM proteins, lipids, transcription factors, and receptors. Currently, the therapeutic mechanism of EVs is based on two main cargoes, RNA (especially miRNA) and proteins (74).

Exosomes have been shown to play an essential role in critical events in the development of liver fibrosis, including hepatocyte injury, immune cell infiltration, HSCs activation, and excessive ECM deposition (75–77). Several animal models of liver disease, including liver fibrosis and drug-induced acute liver injury, have been found to be alleviated by mesenchymal stem cell exosomes (MSCS-Ex) (78–81). For example, AMSC-Ex significantly decreased fiber accumulation, KCs number, and HSCs activation in rats with liver fibrosis. *In vitro*, AMSC-Ex significantly inhibited KC and HSC activation and suppressed the lipopolysaccharide (LPS)/toll-like receptor 4 (TLR4) signalling pathway. By decreasing collagen production and TGF-1 release, MSCs-Ex were also able to reduce the severity of liver fibrosis caused by ClC_4_ (79, 82). MSC-originated exosomes circDIDO1 sponged miR143-3p in HSCs, causing cell cycle arrest, suppression, and apoptosis through promoting PTEN and repressing the ratio of p-AKT/AKT. Furthermore, umbilical cord mesenchymal stem cell exosomes (UCMSC-Ex) boosted the expression of the epithelium-associated marker E-cadherin while decreasing the expression of N-cadherin and vimentin-positive cells, suppressing EMT and preventing hepatocyte apoptosis. Jiang et al. (83) demonstrated that UCMSCs-Ex reduced CCl_4_-mediated hepatocyte injury and liver fibrosis by inhibiting hepatocyte apoptosis and oxidative stress. In addition, MSCs can release immunopotent exosomes, allowing them to exert immunomodulatory effects on the differentiation, activation, and functionality of various subsets of lymphocytes. Tamura et al. (84) found that MSC-derived exosomes increased the production of anti-inflammatory cytokines and the number of T regulatory cells in mice with concanavalin A-induced liver injury, indicating immunosuppressive properties.

The ability of MSCs-Ex to execute drug transport tasks is another therapeutic use for these cells. Recent research has demonstrated that MSCs may package and distribute active medicines *via* their exosomes. These studies pave the way for researching and developing more robust and homing-capable novel medications employing MSCs. Cell-free treatment techniques avoid the potential for carcinogenesis, unneeded differentiation, embolization, cell injection, and infection dissemination associated with MSCs transplantation (21, 80). Furthermore, these therapies are safer, less costly, and more effective. Although MSCs-Ex shows considerable potential for treating liver disease, the absence of a consistent and efficient production process remains a key impediment. Thus, additional studies will be needed to address these important issues.

## 5 Challenges and future directions

Over the past two decades, there has been a rapid increase in cell therapy techniques for treating liver fibrosis. These technologies have accumulated knowledge on enhancing *in vitro* cell manipulation and cell transplantation processes to combat liver fibrosis and enhance liver repair. With the help of cutting-edge technologies such as bioreactors, microfluidics, and 3D bioprinting, these studies are now contributing to the development of new technologies aimed at producing *in vitro* systems that can yield liver-like tissue or whole bioengineered livers (85–87). However, it has been reported that MSCs therapy still has certain limitations for clinical application, including safety, limited cell survival, standardized production, ethical concerns, unavailability of trustworthy animal models, and lack of appropriate injection routes.

First, and perhaps most important, the safety of MSC-based therapy is still under discussion, especially in the context of long-term follow-up. A major concern is the undesired differentiation of transplanted MSCs and their propensity to impair antitumor immune responses and develop new blood vessels, which may contribute to tumor development and spread (88). In addition, neither the administration of MSCs nor their application in clinical studies has been standardized. Stem cells can readily differentiate into other cell types (except myofibroblasts), mediating communication between stem cells and HSCs, hepatocytes, or other intrahepatic cells associated with liver fibrosis, thereby triggering an immune response to promote fibrosis or prevent the reversal of liver fibrosis. Therefore, there is an urgent need for more sizeable prospective validation trials to verify the efficacy and safety of this treatment.

Second, low cell survival and poor integration with host tissue are well-recognized hurdles that confront the field of *in vivo* MSC delivery, and these may be improved by the pretreatment of stem cells (89, 90). Primary stem cells often fail to achieve the desired therapeutic effect due to poor homing ability, a low survival rate, and cellular senescence or decreased viability during *in vitro* culture. Pretreatments include transgenics, hypoxia, inflammatory factors, bioactive compounds, 3D cultures, disease-associated cells, or patient serum. For example, the proliferation of HSCs and collagen deposition in rats with liver fibrosis were more effectively inhibited by HGF gene-transduced MSC (MSCs/HGF) (91). In a separate study, miR122-modified ADMSCs (ADMSC-122) constructed by lentivirus-mediated transfer of miR-122 had a similar effect (92). HGF is a hepatocyte growth factor that promotes hepatocyte regeneration, whereas miR-122 is crucial for inhibiting the proliferation and activation of HSCs. In conjunction with MSCs, they act on intrahepatic cells to treat liver fibrosis and overcome the lack of physiological activity of MSCs cells following transplantation.

Third, the effectiveness and safety of MSCs in treating patients with liver fibrosis have been studied in numerous clinical trials over the last decade. According to National Institutes of Health ClinicalTrials.gov, since January 1, 2012, 44 relevant clinical studies have been registered, 9 of which have been completed. Of these, China (23 cases; 52.3%), Vietnam (4 cases; 9.1%), India (3 cases; 6.8%), Japan (3 cases; 6.8%), South Korea (2 cases; 4.5%), and Belgium (2 cases; 4.5%) are the top six countries. In terms of cell origin, MSCs can be derived from human umbilical cord (14 cases; 31.82%), bone marrow (10 cases; 22.7%), adipose tissue (3 case; 6.8%), teeth (1 case; 3.2%), and unknown (16 cases, 36.4%). Simultaneously, several studies describing the outcomes of clinical trials using MSCs have been published. For instance, Yuwei et al. (93) published a meta-analysis and systematic review of randomized controlled trials (RCTs) to assess the efficacy and safety of MSCs therapy for patients with chronic liver disease. They evaluated 12 RCTs, including 846 patients who met their selection criteria. The results indicated that MSCs improved liver function compared to conventional treatment, primarily in ALB, TBIL, MELD score, and coagulation levels, but there were no significant changes in ALT and AST levels. Furthermore, MSCs treatment could not significantly improve the overall survival rate, with only a slightly positive trend. Five of these studies reported that the only side effect of MSC treatment was fever, with no other serious side effects. In terms of cell source, these clinical trials also show a tendency toward shifting from autologous to allogeneic MSCs. Allogeneic MSCs do not require the painful and complicated process of autologous cells collection (94). In addition, some MSCs can be made into off-the-shelf products due to the absence of immune rejection and the potential for massive expansion. Among these, UCMSC is a promising stem cell that is expected to be produced commercially (95). Many studies have shown that UCMSCs are an ideal source of MSCs because of young cellular age, relative ease of collection, easy batch production, and low allogeneic reactivity (96, 97).

In addition, there are various routes of MSC transplantation, including trans-portal, hepatic artery, peripheral vein transplantation, intrasplenic transplantation, peripheral vein transplantation, intrahepatic and abdominal transplantation, and the respective characteristics of these routes remain unknown. Under identical conditions, MSCs from different transplantation routes have different biodistribution *in vivo*. They may exert their effects on liver fibrosis *via* mechanisms influenced by the microenvironment, resulting in different therapeutic effects on fibrosis. Selecting a suitable stem cell transplantation route to increase the amount of stem cell colonization in liver tissue, enhance cell activity, and maintain the survival time of stem cells in liver tissue may be beneficial to improve the anti-fibrotic effect of MSCs. The indications for different transplantation routes in clinical practice must be further investigated (98, 99).

In contrast to cell-based pretreatment, cell-free therapy is a hot topic for future research. Cell-free strategies have low tumorigenic potential, low preservation costs, and low risk of exogenous infection and thrombosis. Unfortunately, no clinical trial has involved the use of stem cell-derived EVs to treat liver fibrosis and cirrhosis. This phenomenon is primarily due to the lack of standardized methods for extracting large numbers of EVs and the unknown dose and half-life of EVs.

## 6 Conclusion

The immune response is crucial to the genesis and progression of liver fibrosis. Due to their immunomodulatory properties, hepatic differentiation potential, and capacity to produce trophic factors, MSCs and MSC-Ex have emerged as promising agents for treating liver fibrosis. However, many issues need to be addressed before MSCs can be used clinically, including sufficient cell numbers, higher integration efficiency, consistency of *in vitro* and *in vivo* studies, and optimal timing and route of cell transplantation. Therefore, large randomized controlled clinical trials with longer follow-up periods and experimental animal studies are needed to improve the safety and efficacy of MSCs for fibrosis treatment.

## Author contributions

PL: Conceptualization, methodology, investigation, funding acquisition, writing – original draft. YQ: Investigation, writing-original draft. XL: Investigation, methodology. XFZ: Investigation. YL: Conceptualization, supervision, funding acquisition. JX: Conceptualization, supervision, writing-review & editing, funding acquisition. All authors contributed to the article and approved the submitted version.
